# Laser microtextured titanium implant surfaces reduce *in vitro* and *in situ* oral biofilm formation

**DOI:** 10.1371/journal.pone.0202262

**Published:** 2018-09-07

**Authors:** Andrei C. Ionescu, Eugenio Brambilla, Francesco Azzola, Marco Ottobelli, Gaia Pellegrini, Luca A. Francetti

**Affiliations:** Department of Biomedical, Surgical and Dental Sciences, IRCCS Galeazzi Orthopedic Institute, University of Milan, Milan, Italy; VIT University, INDIA

## Abstract

**Introduction:**

Micro- or nano-topography can both provide antimicrobial properties and improve osseointegration of dental implant titanium surfaces. Laser treatment is one of the best surface microtexturing techniques. The aim of this study was to evaluate *in vitro* and *in situ* biofilm formation on a laser-treated titanium surface, comparing it with two conventional surfaces, machined and grit-blasted.

**Methods:**

For the *in vitro* experiment, an oral microcosm biofilm model was developed on the surface of titanium disks and reference human enamel using a bioreactor for 48 h. For the *in situ* experiment, titanium implants with laser-treated, machined and grit-blasted surfaces were mounted on intraoral trays and worn by ten volunteers for 48 h. Biofilm formation was quantitatively evaluated, and surfaces were analyzed using confocal laser scanning microscopy, scanning electron microscopy and energy-dispersive X-ray spectroscopy.

**Results–*in vitro* study:**

Biofilm structures with a prevalence of viable cells covered most of the machined, grit-blasted and human enamel surfaces, whereas less dense biofilm structures with non-confluent microcolonies were observed on the laser-treated titanium. Laser-treated titanium showed the lowest biofilm formation, where microorganisms colonized the edges of the laser-created pits, with very few or no biofilm formation observed inside the pits.

**Results–*in situ* study:**

The biofilm formation pattern observed was similar to that in the *in vitro* experiment. Confocal laser scanning microscopy showed complete coverage of the implant threads, with mostly viable cells in grit-blasted and machined specimens. Unexpectedly, laser-treated specimens showed few dead microbial cells colonizing the bottom of the threads, while an intense colonization was found on the threading sides.

**Conclusion:**

This data suggests that laser-created microtopography can reduce biofilm formation, with a maximum effect when the surface is blasted orthogonally by the laser beam. In this sense the orientation of the laser beam seems to be relevant for the biological interaction with biofilms.

## Introduction

Currently, dental implants are considered the best option for the replacement of missing teeth. In this sense, titanium has long been the most widely used biomaterial, due to its mechanical properties and biocompatibility. [1,2] These characteristics have been furtherly improved using surface treatments that increase roughness and thickness of the surface titanium oxide layer. Surface treatments can also modify the surface energy and wettability of dental implants, having a direct effect on protein adsorption and osseointegration process. These improvements, aimed at facilitating osseointegration, could confer to this therapy option a very high survival rate, reaching 95.7% after 5 years of follow-up and 92.8% after 10 years in healthy patients. [1,3–5]

The infection called peri-implantitis is the most frequent cause of implant failure over time. [6–8] Indeed, approximately 10% of dental implants develop peri-implantitis 5–10 years after implant placement. [9] This pathological condition is caused by a polymicrobial aggressive biofilm colonizing implant and abutment surfaces at the peri-implant crevice level, thus leading to an inflammation of the surrounding soft and hard tissues. [8,10] Removal of biofilm structures adherent to the implant surface allows the regression of the soft-tissue inflammation, [6–8] but this is difficult to achieve due to the high roughness of the implant surface, which is designed to improve osseointegration. Indeed, the increase in surface roughness (SR) facilitates the migration and the retention of pathogenic microorganisms when the surface is exposed, making cleansing procedures very difficult. [6–8,11,12]

Several preventive strategies aimed at controlling biofilm formation on titanium surfaces exposed to the oral environment have been proposed. [13] They are mainly based on the interference with bacterial adherence and colonization using surface immobilized antibacterial molecules, photocatalytic compounds, or more often, by releasing biocidal molecules or metal ions [6,14–18].

A different approach involves the modification of physico-chemical and topographical characteristics of titanium surfaces. High SR and hydrophilicity, albeit to a lesser degree, are known to play a critical role in promoting *in vivo* bacterial adherence and colonization of the surface. [11,13,18] Nevertheless, SR is also critical for the osseointegration process, so the reduction of this parameter may have relevant side effects.

Bioadhesion can also be influenced by surface hydrophilicity. Highly hydrophilic surfaces can promote bacterial adherence [18], yet, since they are surrounded by a monolayer of water molecules, that can prevent bacterial interactions with the surface. [6,11]

Topography is the third parameter able to influence bacterial adherence. Bacterial cells are more rigid than mammalian cells, and therefore are not able to deform to accommodate surface constraints. Therefore, a topographic pattern that promotes adhesion of mammalian cells can usually reduce bacterial adherence. [6,19,20] For these reasons, the creation of micro- or nano-topography patterns on titanium surfaces may be a promising approach, endowing the surface with antimicrobial properties and, at the same time, avoiding the previously described side effects.

Due to the cutting precision at the micro-level and the possibility of modulating the gun parameters, lasers are one of the best and most suitable devices to create a clean and reproducible micropattern on metal surfaces. Laser-modified micro-topography was recently applied to create a new titanium surface for dental implants. This surface showed very positive biocompatibility behavior both *in vitro* and *in vivo*. [21,22] However, very few studies have been performed to date regarding microbial colonization of laser-treated titanium implant surfaces: five studies evaluated *in vitro* titanium bacterial colonization using static monospecific models, while only one study was performed *in situ*. [23–28] From a microbiological point of view, continuous flow bioreactors are considered the best way to reproduce a complex oral microcosm *in vitro*, overcoming the limitations related to the use of simplified static reactors. The aim of this study was to evaluate *in vitro* biofilm formation on a newly designed, laser microtextured titanium surface, using a continuous flow bioreactor and to compare it with that occurring on two conventional surfaces, machined and grit-blasted. Additional aim was to evaluate *in situ* biofilm formation on the same surfaces.

## Materials and methods

### *In vitro* study

#### Specimen preparation

Titanium specimens for the *in vitro* experiment were obtained from Geass srl (Pozzuolo del Friuli, Udine, Italy). A total of 74 grade IV titanium disks (diameter = 6.0 mm, height = 2.0 mm) were obtained and randomly divided into three groups (n = 26). Degrease/cleaning procedures were applied to all titanium specimens by the manufacturer, as follows:

1. rinsing with aqueous alkaline cleaning solution containing nonionic surfactant, to degrease specimens immediately after exiting CNC cutting machine;
2. rinsing with hydrofluoroether methoxynonafluorobutane for degreasing purposes;
3. ultrasonic cleaning in aqueous surfactant solution, then rinsing with ultrapure water.

The first group was left untreated (machined), and its surfaces were not finished or polished, while the other two groups were, respectively, grit-blasted with Al_2_O_3_ particles (25–250 μm) or subjected to laser treatment. Grit- blasting was performed at 400 KPa for 12 s at 65 mm distance of the specimen/implant from the nozzle. Grit-blasted specimens were additionally subjected to steps 2 and 3 of cleaning treatment after grit-blasting.

The laser-treated surfaces were obtained by a controlled micro-ablation using a pulsed, Nd: YAG source diode pumped solid state (DPSS) laser (355 nm wavelength), in a Q-Switch output. Emission in the Q-Switch mode allowed the generation of brief impulses (tenths of nanoseconds), which removed the material from the surface. This operation mode was claimed to assure a high repeatability of the process, without thermally altering surrounding areas, avoiding cluster formation and any contamination of the surface by elements other than titanium. [29] The laser beam power was set to generate hemispheric pits of 20 μm diameter, whereas the scanning motion and pulse frequency were set to generate pits of 30 μm pitch. In this way, each titanium disk was laser-microtextured in 52 s. Laser-treated specimens were additionally subjected to step 3 of cleaning treatment after laser microtexturing.

An additional group of human enamel disks was used as a reference. The Institutional Review Board of the University of Milan approved the use of the tooth disks, and written informed consent was obtained from the donors. Twenty-six enamel disks (diameter = 6.0 mm, height = 2.0 mm) were obtained from anterior human teeth, extracted for orthodontic reasons (Oral Surgery Unit, Department of Biomedical, Surgical and Dental Sciences, Milan, Italy) using a water-cooled trephine diamond bur (INDIAM, Carrara, MS, Italy). [30] Disks were then polished using 1000 and 4000-grit grinding paper (Buehler, Lake Bluff, IL, USA) mounted on a polishing machine (Motopol 8; Buehler, Düsseldorf, Germany).

#### Surface roughness

The SR of the specimens was measured on 12 disks/group using a profilometer (Sutronic 3+; Taylor Hobson, Leicester, UK). A distance of 1.75 mm was measured in three line scans perpendicular to the expected grinding grooves for each specimen, using a standard diamond tip (tip radius 2 μm, tip angle 90°) and a cut-off level of 0.25. Data were expressed as Ra.

#### Surface free energy (SFE)

**Fig 1:**
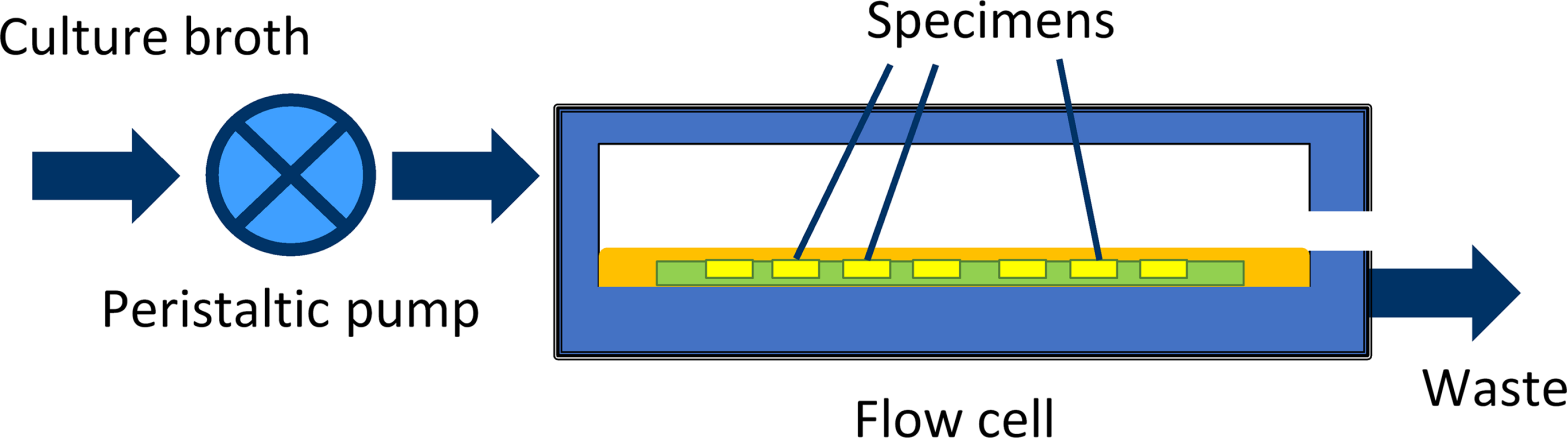
MDFR functioning diagram. A peristaltic pump is used to obtain a continuous flow of nutrients over the surface of the tested specimens. Culture broth is then discarded.

Contact angles between the surface of the various materials and ultrapure, HPLC-grade water were determined using the sessile drop method and a computer software for contact angle measurement (Surftens 47, OEG Gesellschaft für Optik, Elektronik & Gerätetechnik mbH, Frankfurt, D). A 6 μl drop was placed on each of seven randomly selected specimens for each material (Fig 1). Left and right contact angles were averaged, and the surface free energy was calculated according to the formula:

$$\cos\;Ɵ=-1+2\;\left(\frac{\gamma_{sv}}{\gamma_{lv}}\right)$$

considering that the total surface free energy of water (γ_lv_) at the temperature at which experiments were performed (20°C) is 72.8 mJ/m^2^.

#### Saliva collection

To create an oral microcosm inoculum, paraffin-stimulated whole saliva was obtained from five healthy donors. The Institutional Review Board of the University of Milan approved the use of saliva, and written, informed consent was obtained from the donors. They refrained from oral hygiene for 24 h, had no active dental disease, and did not use antibiotics for at least 3 months. The saliva was obtained within 30 min immediately prior to the beginning of the experiment and pooled.

#### Microbiological procedures

Culture media were obtained from Becton-Dickinson (BD Diagnostics-Difco, Franklin Lakes, NJ, U.S.A.), and reagents were obtained from Sigma-Aldrich (Sigma-Aldrich, St. Louis, MO, U.S.A.).

Prior to microbiological procedures, all specimens were placed into polystyrene multi-well plates and sterilized with a chemical peroxide-ion plasma sterilizer (STERRAD, ASP, Irvine, CA, USA). The chemiclave reached a maximum temperature of 45°C, thus preventing possible heat-related damage of the specimens.

The drip flow reactor (MDFR) used in the study was a modification of a commercially available Drip Flow Reactor (DFR 110; BioSurface Technologies, Bozeman, MT, USA). The modified design allowed the placement of customized specimen trays on the bottom of the flow cells and the complete immersion of the specimens into the surrounding flowing medium (Fig 1). [31] After sterilization with STERRAD, the MDFR was assembled into a sterile hood. The specimens were then inserted into PTFE molds placed on the bottom of the flow cells. A total of 10 ml of saliva inoculum was placed into each flow cell and the MDFR was then incubated at 37°C for 4 h to allow the formation of a salivary pellicle on the surface of the specimens and bacterial adherence. Then, a constant flow of sterile modified artificial saliva medium [32] including 2.5 g/L mucin (type II, porcine gastric), 2.0 g/L bacteriological peptone, 2.0 g/L tryptone, 1.0 g/L yeast extract, 0.35 g/L NaCl, 0.2 g/L KCl, 0.2 g/L CaCl_2_, 0.1 g/L cysteine HCl, 0.001 g/L hemin, and 0.0002 g/L vitamin K_1_ was provided by a peristaltic pump at a flow rate of 9.6 mL/h. The MDFR was operated for 48 h to allow the development of a multilayer biofilm on the surfaces of the specimens. At the end of the incubation the flow was stopped, and the trays were extracted from the flow cells. The specimens were carefully removed from the trays using a pair of sterile tweezers and gently rinsed with sterile phosphate-buffered saline (PBS) at 37°C to remove non-adherent cells. The specimens were then placed into sterile 48-well plates.

#### Viable biomass assay

Viable and metabolically active biomass adherent to the specimens’ surface was assessed using a tetrazolium-based assay as described previously. [33] Briefly, MTT stock solution was prepared by dissolving 5 mg/mL 3-(4,5)-dimethylthiazol-2-yl-2,5-diphenyl-tetrazolium bromide in sterile PBS and PMS stock solution was prepared by dissolving 0.3 mg/mL of N-methylphenazinium methyl sulfate in sterile PBS. The solutions were stored at 2°C in light-proof vials until the day of the experiment, when a fresh measurement solution (FMS) was prepared by diluting 1:10 v/v of MTT stock solution and 1:10 v/v of PMS stock solution in sterile PBS. A lysing solution (LS) was prepared by dissolving 10% v/v of sodium dodecyl sulfate and 50% v/v dimethylformamide in deionized water.

A total of 300 μL of FMS were added to each well immediately after positioning the specimens (n = 14/group), and the plates were incubated for 3 h at 37°C under light-proof conditions. During incubation, electron transport across the microbial plasma membrane and, to a lesser extent, microbial redox systems, converted the yellow salt to insoluble purple formazan crystals. The conversion at the cell membrane level was facilitated by the intermediate electron acceptor (PMS). The unreacted FMS was gently removed by aspiration, and the formazan crystals were dissolved by adding 300 μL of LS to each well. The plates were stored for an additional 1 h under light-proof conditions at room temperature; 100 μL of the solution was then transferred into 96-well plates. The absorbance of the solution was measured using a spectrophotometer (Genesys 10-S, Thermo Spectronic, Rochester, NY, USA) at a wavelength of 550 nm; results were expressed as relative absorbance in optical density (OD) units corresponding to the amount of adherent, viable and metabolically active biomass.

#### SEM analysis

The specimen surface was observed before and after biofilm formation using scanning electron microscopy (SEM). A set of specimens (n = 3/group) not used for microbiological procedures was mounted on stubs with conductive tape, sputter coated (JEOL FFC-1100, Japan), and observed with SEM (Tabletop SEM TM3030 Plus, Hitachi, Schaumburg, IL, USA) at 15 KV acceleration voltage. Four randomly selected fields at two different magnifications (500X and 5000X) were recorded for each specimen.

Specimens undergoing SEM analysis after biofilm formation (n = 3/group) were placed into a cacodylate-buffered 2% glutaraldehyde fixative solution (pH = 7.4) for 48 h. The specimens were then passed through a graded ethanol series (50, 70, 80, 85, 90, 95, and 100%, v/v). Finally, they were subjected to critical point drying (Critical-Point Dryer, EMS 850, Hatfield, PA, USA), mounted on stubs with conductive tape, sputter coated, and observed with SEM at 15 KV acceleration voltage. Four randomly selected fields at two different magnifications (500X and 5000X) were recorded for each specimen.

#### Energy-dispersive X-ray spectroscopy (EDS)

An additional set of specimens (n = 3/group) was analyzed using EDS probe (SwiftED3000, Oxford Instruments Analytical Ltd., Abingdon, Oxfordshire, UK) before and after biofilm formation. The specimens were processed as previously described, without sputter coating. Three randomly selected 300 x 300 μm fields were analyzed for each specimen in full-frame mode using an acquisition time of 150 s at 15 KV accelerating voltage in a surface-charge reduction mode (low vacuum). Acquired data represented the elemental composition of the 1 μm-thick superficial layer from which electrons were extracted by the accelerated beam.

#### Confocal laser scanning microscopy (CLSM)

Specimens undergoing CLSM analysis (n = 3/group) were stained using the FilmTracer™ LIVE/DEAD® Biofilm Viability Kit (Invitrogen Ltd., Paisley, UK), then observed using a confocal laser scanning microscope (Leica TCS SP2, Leica Microsystems, Wetzlar, DE). Confocal stacks were obtained using a dry 10X (NA = 0.7) objective and digitalized using the Leica Application Suite Advanced Fluorescence Software (LAS-AF, Leica microsystems) at a resolution of 2048 x 2048 pixels with a zoom factor of 1.0 and a scan speed of 400 Hz. Specimens were excited at a wavelength of 488 nm. Emission was acquired at 500–570 nm (green channel, live bacteria) and 610–760 nm (red channel, dead bacteria). Four randomly selected image stack sections were recorded for each specimen and maximum intensity projections (MIP) were obtained. For each image stack section, 3D-rendering reconstructions were obtained using Drishti (Ajay Limaye, Australian National University, CAN, AUS http://sf.anu.edu.au/Vizlab/drishti/). MIP images, splitted into green (live bacteria) and red (dead bacteria) channels, were analyzed using COMSTAT 2.1 software running on ImageJ platform to determine the percentage of biomass surface coverage, and the amount of live and dead bacterial cells in the biofilms.

### *In situ* study

#### Specimen preparation

Dental implants for *in situ* experiments were obtained from Geass srl (Pozzuolo del Friuli, Udine, Italy). A total of ten dental implants (Implant model “Milano way”, diameter = 4.0 mm, length = 9.0 mm,) for each surface treatment group (machined, grit-blasted and laser-treated) were prepared by the manufacturer as previously described in the *in vitro study*. The tested implant type was laser-microtextured in 136 s. Implants were sterilized with gamma irradiation and provided by the manufacturer in sterile packaging. The implants were separated into halves parallel to the long axis, with a random longitudinal orientation, using a diamond separating disk (1UM2002Z, Horico Dental GmbH, Berlin, Germany) under constant water cooling. Then, specimens were cleaned using an ultrasonic bath for 30 min in distilled water, rinsed with absolute ethanol, and stored dry until use.

#### Study design

The study was designed as a randomized, controlled, double-blind trial, performed according to the principles of the Declaration of Helsinki updated by the World Medical Association in 2013. [34] The Institutional Review Board of the University of Milan approved the protocol of the *in situ* study (codename: SALTiBO-2017).

#### Volunteers

According to the *in vitro* study data, the sample size was determined considering the subjects as the primary source of variability, an OD (adherent viable biomass) standard deviation of 0.198, an α value of 0.05 and a power of 0.8. A total of ten subjects were estimated to be necessary, and were subsequently recruited (seven women, three men; aged 20–32 years old with a mean age of 22.5 years).

Inclusion criteria were absence of systemic antibiotic therapy in the last 3 months; full dentition (complete dental formula); absence of caries, and plaque index < 10%; [35]; absence of periodontal disease or inflammatory reactions of the oral soft tissues, and bleeding index < 10%; [36]; non-smokers for at least 10 years prior to the beginning of the experimentation. [37] Before obtaining written, informed consent, all subjects eligible for the study received extensive verbal and written information regarding all procedures.

#### Trays

**Fig 2:**
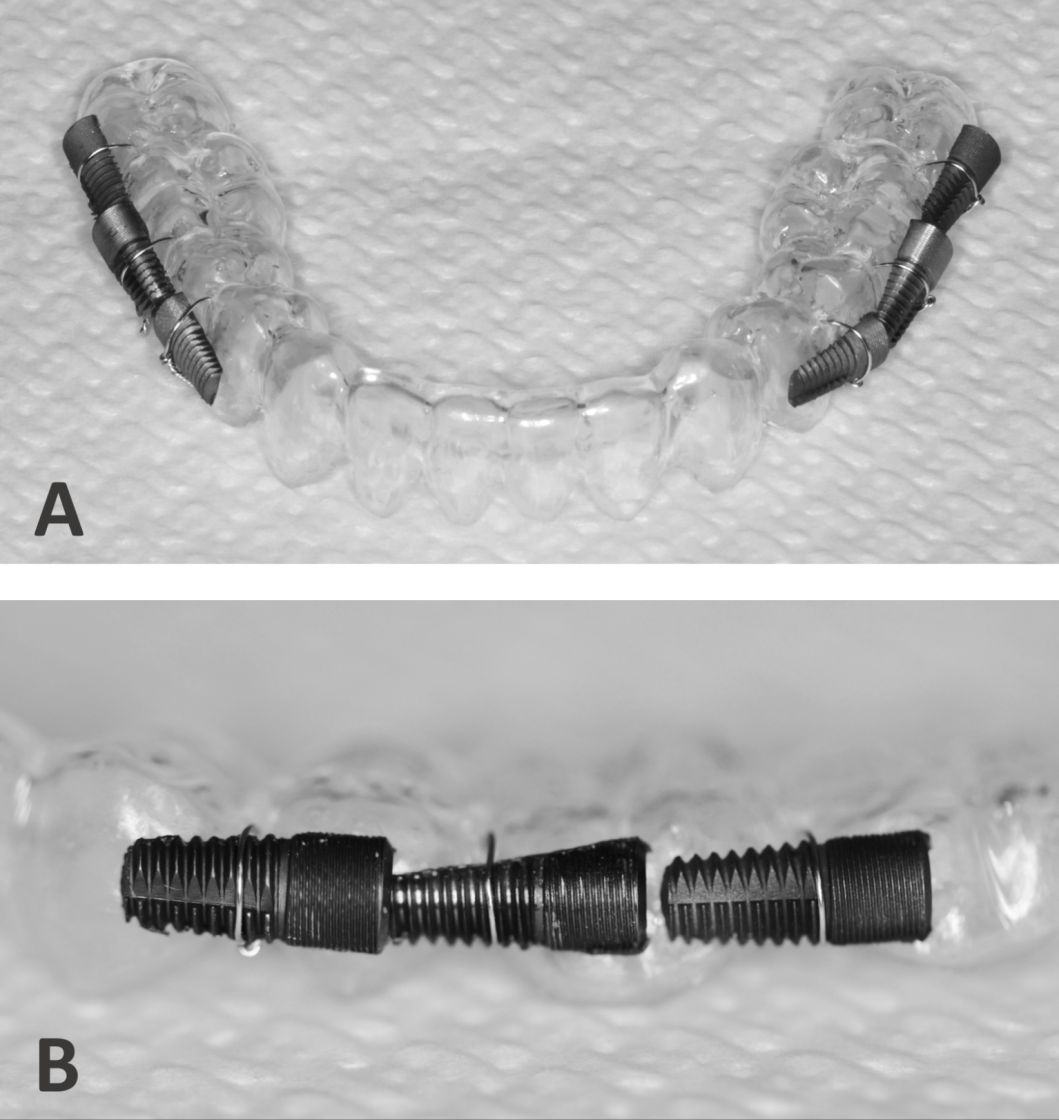
Intraoral trays. A: Individual mandibular thermoformed acrylic customized tray obtained using a study cast of the lower jaw. B: On the buccal part of the device three half-implants, one for each experimental surface, were fixed horizontally on each side (three on the right and three on the left) using orthodontic 0.1 mm wire.

An individual mandibular thermoformed acrylic customized tray was obtained from the dental laboratory for each subject, using a study cast of the lower jaw. On the buccal part of the device, corresponding to the molar/premolar region bilaterally, three half-implants, one for each experimental surface, were fixed horizontally on each side (three on the right and three on the left) using orthodontic 0.1 mm wire (Fig 2). Subjects were identified with their initials, and the position of the implant specimens on the tray was determined using a randomization table. All trays were then sterilized using the chemical peroxide-ion plasma sterilizer.

#### In situ experiment

All subjects received a professional tooth cleaning and detailed instructions on the handling of the trays one week prior to the start of the experiment. Subjects were asked to refrain from using mouthwashes and chewing-gum one month before the beginning of the experiment and for the duration of it. The subjects were asked to wear the tray for a total of 48 h. During this period, they were asked not to alter their usual diet and not to take any drugs. The tray was removed and stored in PBS solution only during oral hygiene procedures and meals. The tray was not rinsed or cleaned in any way. During the whole period, subjects were also asked to brush their teeth twice a day with a fluoride-containing toothpaste (1400 ppm F^-^).

Subjects were informed that they were free to stop and exit the study anytime. Subjects and those performing the technical procedures were blinded to the surfaces tested, and the specimens’ position on the trays (double-blind model).

After 48 h, trays were removed and immediately placed in sterile PBS at 37°C. The specimens were carefully removed from the trays using sterile tweezers and cutting pliers, transferred into 24-well plates and processed for MTT assay (n = 10 specimens/group), SEM observation (n = 5 specimens/group) and CLSM analysis (n = 5 specimens/group), as previously described.

### Statistical analysis

Statistical software (JMP 10.0, SAS Institute, Cary, NC, USA) was used to analyze raw data. Shapiro-Wilk’s test was used to preliminarily check the normality of the data sets’ distribution, and Bartlett’s test was used to check homogeneity of variances. One-way ANOVA and Student’s post-hoc t-test were used to highlight significant differences between groups, at a level of significance (α) of 0.05. Since the roughness data did not yield a normal distribution, even after log transformation, nonparametric analysis was performed using the Wilcoxon method to compare differences between groups. For this reason, the same nonparametric analysis was applied to analyze SFE and CLSM data.

## Results

### *In vitro* experiment

**Fig 3:**
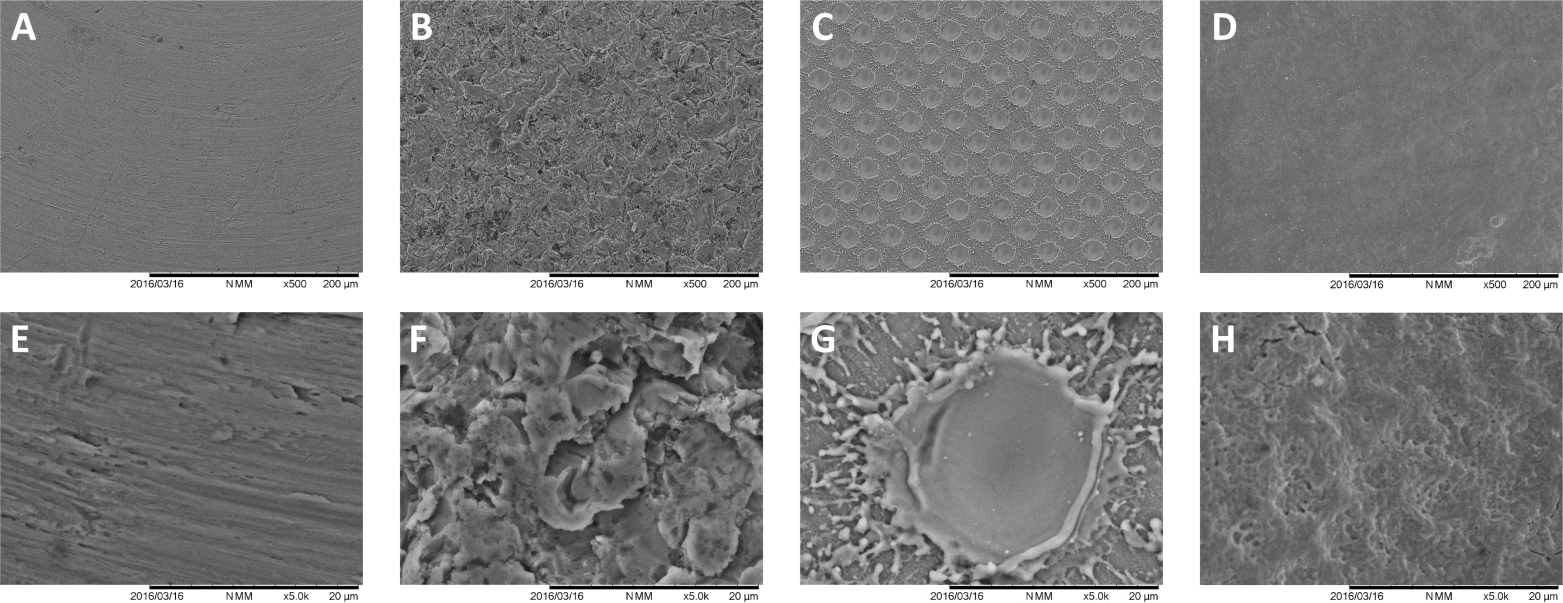
SEM analysis of the *in vitro* tested surfaces prior to microbiological procedures. A, B, C, and D, show 500X magnifications of machined, grit-blasted, laser-treated and enamel surfaces. E, F, G, and H, show the same surfaces at 5000X magnification. Grooves were present on the machined titanium surfaces, grit-blasted specimens showed characteristic surfaces with high SR, and laser-treatment left very regular microtextured surface with pits of 18–20 μm diameter.

The analysis of the tested surfaces before microbiological procedures is shown in Fig 3. The surface of the specimens displayed specific morphological characteristics for each group, such as grooves shown by the machined titanium surfaces, high SR due to grit-blasting procedures and the very regular pits left by laser treatment (18–20 μm diameter). At high magnification (5000X), laser-created pits had a very smooth inner surface combined with a surface between pits showing titanium debris caused by the laser blast.

SR analysis of the tested surfaces before microbiological procedures is shown in Table 1. As expected, grit-blasting of titanium surfaces produced the highest roughness (p < 0.0001), while enamel surfaces, due to the polishing procedures, showed the least roughness (p < 0.0001). Laser-modification of the titanium surface micro-topography yielded significantly higher roughness than the untreated, machined surfaces (p = 0.0004), and a lower roughness than those of the grit-blasted (p < 0.0001).

**Table 1:** Surface roughness (Ra) and total surface free energy of the tested titanium surfaces and reference surface (human enamel).

Group	Mean Ra	Standard Deviation	Standard Error	Total SFE	Standard Deviation	Standard Error
Machined	0.3533^c^	0.1376	0.0397	42.8289^b^	2.4934	0.8311
Grit-blasted	0.8416^a^	0.0741	0.0214	23.9220^c^	2.1092	0.9432
Laser-treated	0.6167^b^	0.0692	0.0200	12.3433^d^	1.7671	0.7214
Enamel (reference)	0.1908^d^	0.0817	0.0236	56.2971^a^	7.4716	2.824

Mean, standard deviation and standard error are indicated for each group. Different superscript letters indicate statistically significant differences between groups (Wilcoxon nonparametric post-hoc method).

**Fig 4:**
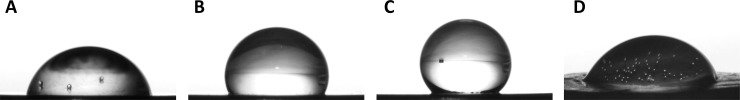
Sessile drop method for SFE evaluation. A 6 μl drop of HPLC-grade ultrapure water was photographed (EOS 500D camera, EF 100mm 2.8f macro lens and Speedlite 470EX bounced flash, Canon, Tokio, Japan) on the surfaces of the experimental specimens as follows: A, machined; B, grit-blasted; C, laser-treated; D, enamel. The hydrophobic behavior of grit-blasted and laser microtextured titanium surfaces can be clearly seen compared to machined ones. The hydrophobic behavior of laser-treated surfaces is likely due to a lotus leaf effect. Hydrophilic enamel reference surfaces are also shown.

SFE results (Table 1 and Fig 4) showed that grit-blasting procedures significantly decreased SFE when compared to machined surfaces (p<0.0001), and laser-microtexturing significantly decreased SFE when compared to grit-blasting (p<0.0010). Enamel showed the significantly highest SFE.

**Fig 5:**
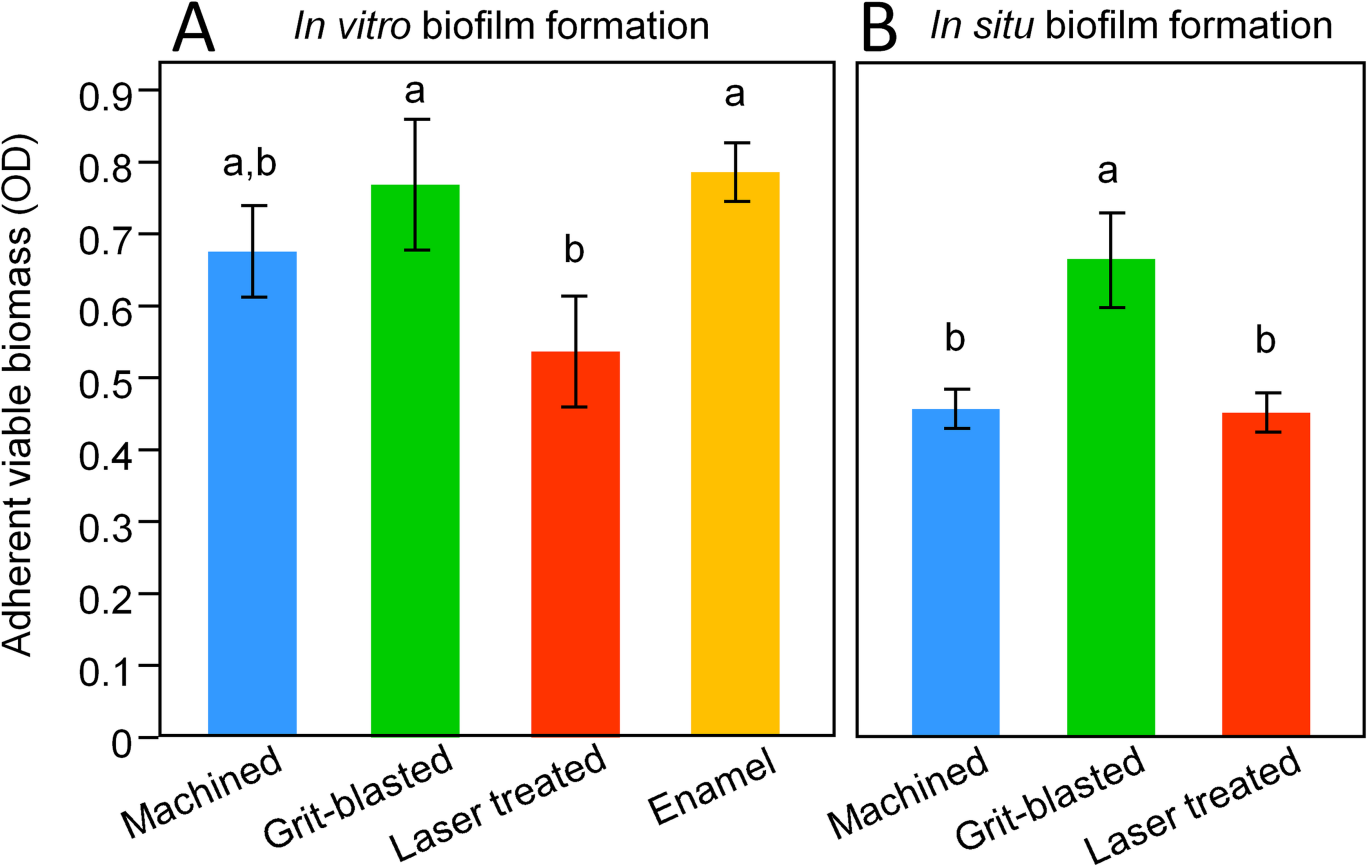
Biofilm formation, assessed by MTT assay. Data are shown as means +/- 1 SD. In each study, different superscript letters highlight significant differences between groups (p<0.05). A: *in vitro* data showed that laser-modified surfaces were less colonized than grit-blasted ones. Machined surfaces showed intermediate values of biofilm formation, non-significantly different from grit-blasted or laser-treated surfaces. Enamel reference surfaces showed similar biofilm formation as grit-blasted surfaces. B: machined and laser-treated surfaces were less colonized than grit-blasted ones, while no significant differences were identified between machined and laser-treated surfaces.

Biofilm formation, assessed by MTT assay (Fig 5A), showed that laser-modified surfaces were significantly less colonized than grit-blasted surfaces (p = 0.0251). Machined surfaces showed intermediate values of biofilm formation, non-significantly different from grit-blasted or laser-treated surfaces (p = 0.3397 and p = 0.1852, respectively). Enamel reference surfaces showed similar biofilm formation as grit-blasted surfaces.

**Fig 6:**
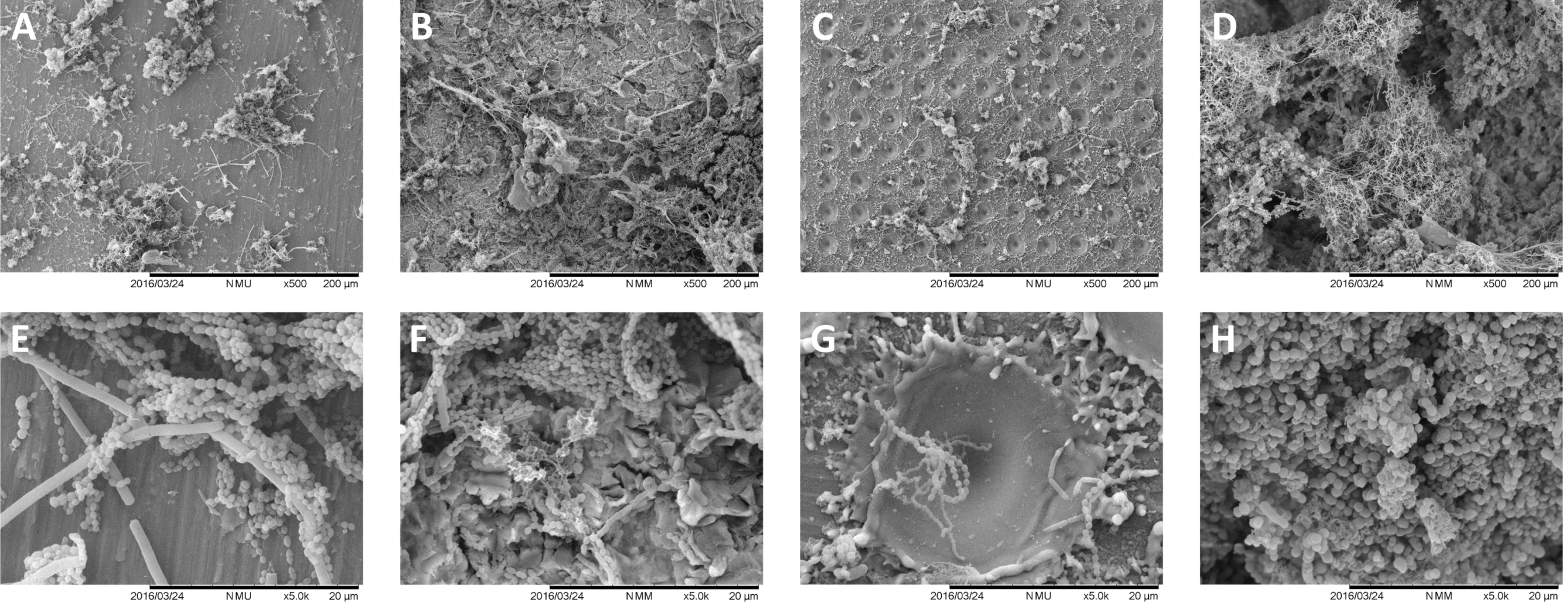
SEM analysis of the tested surfaces after *in vitro* biofilm formation. A, B, C, and D, show 500X magnifications of machined, grit-blasted, laser-treated and enamel surfaces. E, F, G, and H, show the same surfaces at 5000X magnification. Grit-blasted and enamel specimens showed complete surface coverage by a multi-layered biofilm. Machined surfaces showed large microcolonies covering a minor part of the surface, and laser-treated surfaces showed the least biofilm formation.

**Fig 7:**
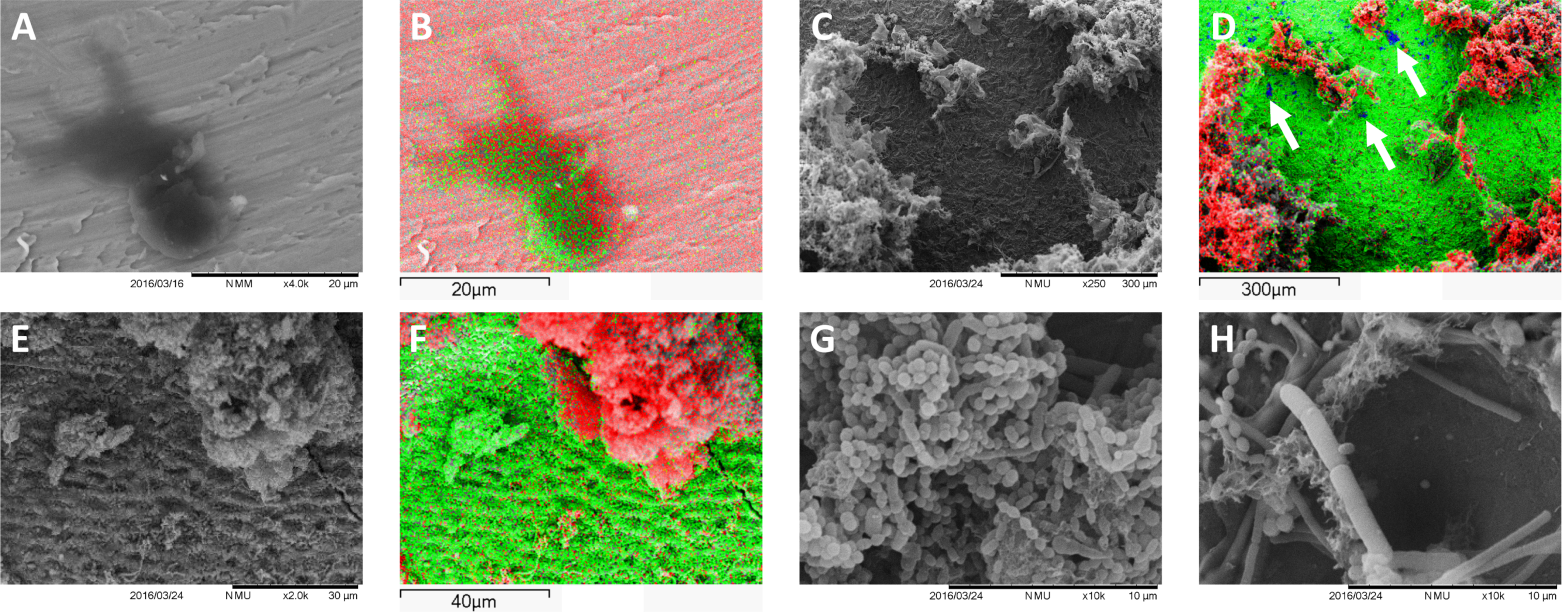
SEM and acquired elemental maps from EDS analysis. A, B: machined surfaces (titanium displayed in red) showed sparse, dark-gray islets made of carbon (in green) that can be regarded as residues of the machining procedures, incompletely removed by subsequent cleansing. C, D: grit-blasted specimens examined after biofilm formation (highlighted by carbon presence, in red) showed aluminum traces (blue, arrows) corresponding to alumina particles embedded into the titanium surface (green) and still present after biofilm formation. E, F: Enamel surfaces (calcium in green) covered by biofilm structures (carbon in red). No contaminating agents derived from polishing procedures can be observed. G, H: high magnification of grit-blasted and laser-treated specimens, respectively, after biofilm formation. In laser-treated specimens, microorganisms show a colonization pattern where cocci, bacilli and extracellular matrix residues can be found on the edges of the pits. Reduced or no biofilm formation can be seen in the inner part of the pits, which were mostly colonized by streptococcal forms.

SEM analysis of the surfaces (Figs 6 and 7) confirmed the findings of the MTT assay, with the grit-blasted and enamel surfaces completely covered by a multi-layered biofilm. Machined surfaces showed relatively large microcolonies covering a small part of the surface, with laser-treated surfaces displaying the least biofilm formation. At high magnification (Figs 6G and 7H), microorganisms show a colonization pattern where cocci, bacilli and extracellular matrix residues can be found on the edges of the pits. Reduced or absent biofilm formation was found in the inner part of the pits, which were mostly colonized by streptococci.

EDS analysis and acquired elemental maps (Fig 7B, 7D and 7F) showed that machined surfaces displayed sparse, dark-gray islets made of carbon (Fig 7B). Since these structures were found on pristine specimen surfaces, they can be regarded as residues of the machining procedures incompletely removed by subsequent cleaning. Grit-blasted specimens showed aluminum traces corresponding to alumina particles embedded in the surface and still present after biofilm formation (Fig 7D, arrows). Laser-treated surfaces displayed a titanium surface without any contamination. Enamel surface composition included Ca, O, and P, without contaminating agents derived from the polishing procedures.

**Fig 8:**
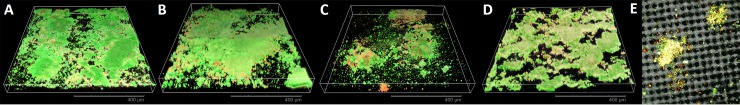
CLSM reconstructions of *in vitro* biofilm formation. A, B, C, and D, show 3D reconstructions of machined, grit-blasted, laser-treated and enamel surfaces. E shows a MIP of laser-treated surface, where the titanium surface was acquired on a separate channel and displayed in gray. In all reconstructions, viable microorganisms are stained in green, while dead microorganisms are stained in red. Compact biofilm structures with a prevalence of viable cells covering most of the machined, grit-blasted and enamel specimens can be observed. Laser-treated surfaces displayed less dense biofilm structures mainly constituted by microcolonies of approximately 200 μm diameter.

CLSM reconstructions (Fig 8) showed compact biofilm structures with a prevalence of viable cells covering most of the machined, grit-blasted and enamel specimens. Laser-treated surfaces had a different morphological appearance, showing less dense biofilm structures with a prevalence of microcolonies approximately 200 μm in diameter. Biofilm structures showed significantly lower surface coverage of laser-treated surfaces than grit-blasted and enamel ones (Table 2, p<0.0001). No significant difference between tested surfaces were highlighted in terms of percentage of viable and dead bacteria.

**Table 2:** Calculations of total surface coverage, and percentage of viable and dead bacteria in the biomass, resulting from CLSM MIP images.

Group	% surface coverage	% Live / Total	% Dead / Total
machined	68.71(9.50)^a,b^	59.50(3.17)a	40.50(3.17)a
grit-blasted	81.40(16.29)^a^	61.75(2,97)a	38.25(2.97)a
laser-treated	52.53(14.34)^b^	61.19(3.06)a	38.81(3.07)a
enamel (reference)	86.55(16.54)^a^	62.94(1.53)a	37.06(1.53)a

Means (+/- 1 standard deviation) are indicated for each group. Different superscript letters indicate statistically significant differences between groups (Wilcoxon nonparametric post-hoc method).

#### *In situ* experiment

MTT assay results are displayed in Fig 5B. Since data were not normally distributed, they were log-transformed prior to statistical analysis (Shapiro-Wilk’s test after log transformation: p = 0.1844, Bartlett’s test: p = 0.3237). The results showed that machined and laser-treated surfaces were significantly less colonized than grit-blasted surfaces (p = 0.0025 and p = 0.0021, respectively). No significant differences were identified between machined and laser-treated surfaces (p = 0.9553).

**Fig 9:**
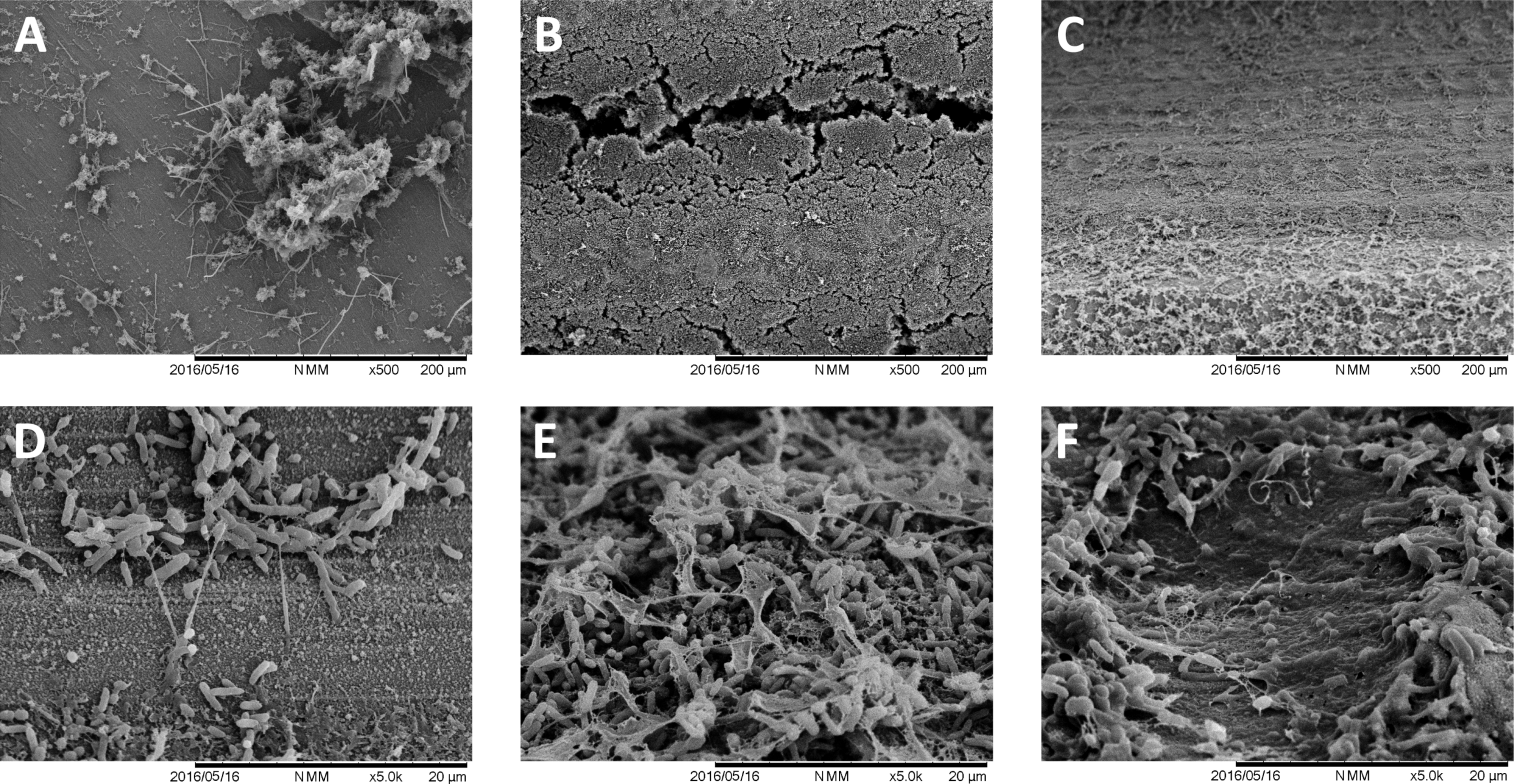
SEM analysis of the tested surfaces after *in situ* biofilm formation. A, B, and C, show 500X magnifications of machined, grit-blasted, and laser-treated surfaces, respectively. E, F, and G, show the same surfaces at 5000X magnification. A similar biofilm formation pattern as in the *in vitro* experiment can be observed.

SEM analysis (Fig 9) confirmed the biofilm formation data, showing a similar biofilm formation pattern as in the *in vitro* experiment. A very high biofilm formation was observed over grit-blasted specimens, completely covering the specimens’ surfaces. Machined surfaces were covered by microcolonies, and laser-treated surfaces showed microbial colonization concentrated on the pit margins, with very few cells, mainly cocci, adherent to the inner parts of the pits.

**Fig 10:**
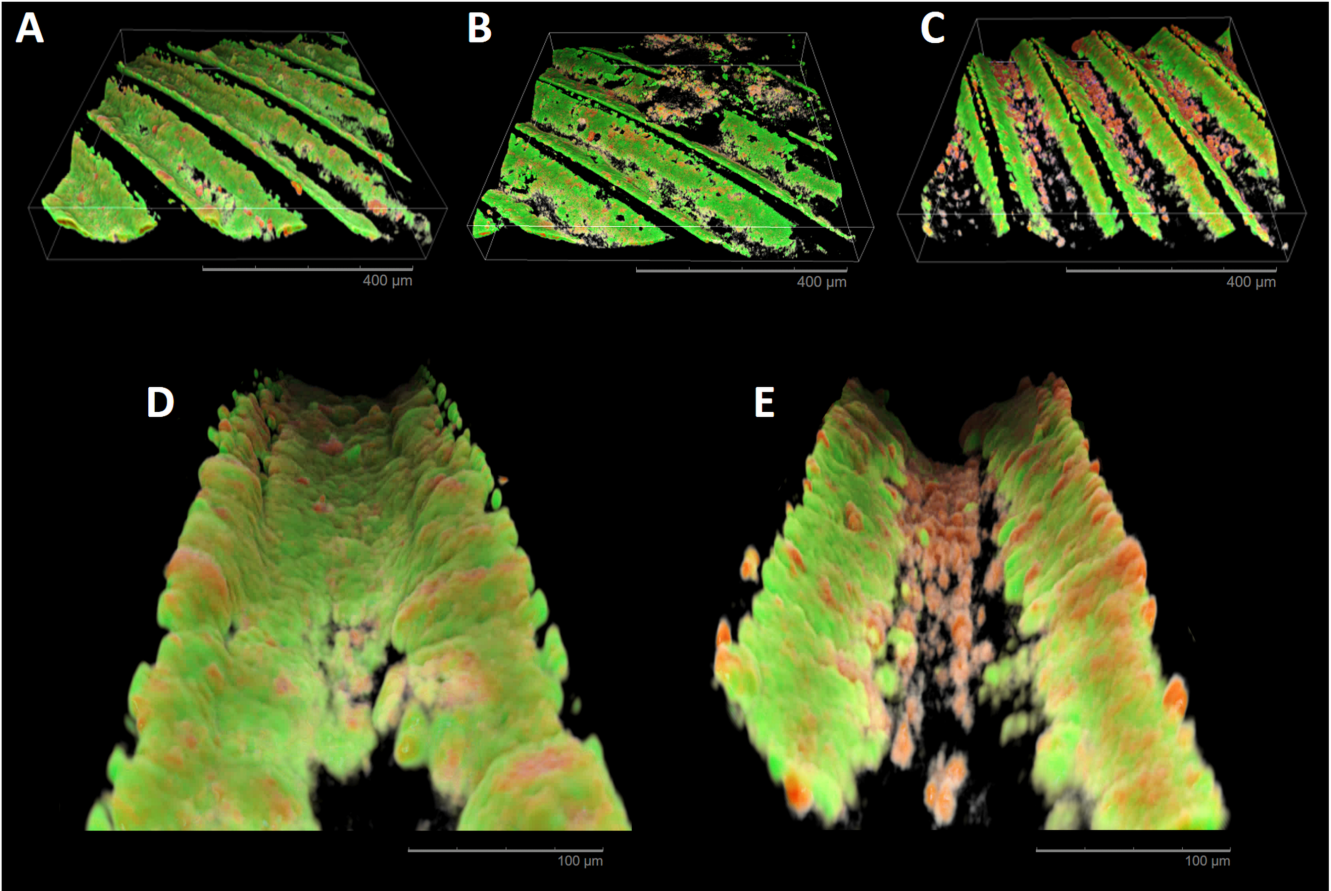
CLSM reconstructions of *in situ* biofilm formation. A, B, and C, show 3D reconstructions of machined, grit-blasted, and laser-treated surfaces, respectively. A complete coverage of the inner parts of the implant threads with mostly viable cells can be observed in all specimens except for laser-treated surfaces. D (grit-blasted specimen) and E (laser-treated specimen) reconstructions show at a higher magnification the inner part of an implant thread. Laser-treated specimens present few dead microbial cells colonizing the bottom of the threads, while an intense colonization of predominantly viable cells can be found on the inclined surfaces of the threads.

CLSM analysis (Fig 10) showed a complete coverage of the inner parts of the implant threads with mostly viable cells in all specimens, except for laser-treated. Surprisingly, laser-treated specimens (Fig 10E) showed a section with few dead microbial cells colonizing the bottom of the threads, while an intense colonization of predominantly viable cells was found on the threading sides. This morphology was different from in vitro CLSM results.

## Discussion

Several *in vitro* and *in vivo* studies demonstrated that micron-scale and submicron-scale structural features of dental implant surfaces can increase osteoblast differentiation and peri-implant bone formation. Increasing surface hydrophilicity can have the same effect [3–5]. Nevertheless, an increase in SR and hydrophilicity seems to facilitate biofilm formation, although this is still under debate. [11,13,18–20,38–40] Teughels and coworkers conduced a systematic review in 2006 to evaluate the influence of surface characteristics (roughness, hydrophilicity and chemistry) on supragingival and, to a lesser extent, subgingival biofilm formation. [18] They concluded that both an increase in surface roughness (above a Ra threshold of 0.2 μm, as previously found by Bollen and coworkers [41]) and in hydrophilicity facilitates biofilm formation on transmucosal implant surfaces, including titanium, and that surface roughness had a predominant effect over hydrophilicity. Schwarz and coworkers in 2007 investigated the effects of SR and hydrophobicity of titanium implant surface on initial supragingival biofilm formation. [39] In agreement with Teughel and coworkers, they concluded that early stages of biofilm formation were more influenced by SR than by hydrophobicity of the surface. Truong and coworkers in 2010 studied the effect of titanium SR on bacterial adherence, [38] demonstrating that only roughness on the nanoscale (and not on a micrometer scale) is able to influence bacterial interactions with the surface. They concluded that SR is the most important factor influencing bacterial adherence, and that other interfacial parameters had little or no relevance. It must be pointed out that little or no data are available in literature about the influence of surface characteristics on biofilm formation, the next step in the bacterial colonization process.

Our data show that there is no correlation between surface roughness and *in vitro* or *in situ* biofilm formation. In fact, all tested surfaces in the present study showed roughness values higher or in the range of the 0.2 μm Ra threshold. One would have expected higher biofilm formation on laser-treated surfaces, since they were twice as rough as the machined ones, and lower biofilm formation on enamel surfaces that showed surface roughness four times lower than grit-blasted titanium surfaces. Furthermore, no correlation was found between surface free energy and *in vitro* or *in situ* biofilm formation. As also demonstrated in a recent study on resin composite materials [42], this discrepancy shows to what extent other factors, such as surface chemistry or topographical modifications of the surface, may have a major role in modulating biofilm formation.

While SR and SFE are intrinsic properties of the surface, surface topography (ST) is a user-defined feature of the surface that can considerably change the physicochemical properties of different materials, including SR and hydrophobicity. [19] As also demonstrated in the present study, introducing a particular ST can considerably change the hydrophobicity of a surface. This has been found to reduce bacterial adherence. [43] Experimental data suggest that there may be optimal topographical features to reduce bacterial adherence, even if relevant inter-species differences are reasonably expected. [19,44]

There are many techniques available for creating micro- and nano-patterned topography. This can be achieved either by material addition or subtraction to the surface. Both can be obtained using masks, which allow processing of the whole surface, for example stereolithography or anodic oxidation, or by a sequential process such as electron beam lithography or laser treatment. In particular, laser surface texturing allows the creation of a wide range of surface micro- and nano patterns on very different materials [45], including metallic, ceramic, and polymeric biomaterials. [26] This technique shows relevant advantages in terms of flexibility, simplicity, and reproducibility, representing a very promising option for improvement of biological properties of biomaterials’ surfaces.

In this study, the biofilm formation on a laser-treated titanium surface was compared with a conventional grit-blasted titanium surface and a conventional machined titanium surface. This surface was characterized by regularly spaced 20 μm-diameter circular holes with a concave bottom and a 30 μm distance between the center of the holes. This geometry is created using laser ablation. The *in vitro* data on flat titanium disks demonstrated that biofilm formation on laser-treated surfaces was significantly lower than grit-blasted surfaces, but not significantly lower than machined surfaces. CLSM images (Fig 6) and SEM images (Figs 4 and 5) showed less organized biofilm structures covering a smaller part of the specimens’ surface. Data from *in situ* study showed a similar pattern of biofilm formation. As the *in situ* study tested dental implant screw surfaces, it was possible to assess the bacterial colonization pattern in a similar way to *in vivo* conditions. CLSM images (Fig 8) show a dense biofilm structure covering the threading sides. The prevalence of dead cells on the biofilm covering the bottom of the threads suggests that the presence of the laser pits on the surface can have a role in reducing biofilm formation. This effect is maximized when the surface is blasted orthogonally by the laser beam, when the beam blasts the titanium surface at a different angle, as on the inclined portion of the threads, this effect is lost. The almost complete absence of biofilm formation of the top of the threads can be explained by the contact of this area with the oral mucosa.

In the literature, only three *in vitro* and one *in situ* studies considered the microbiological behavior of the laser-treated surface. Cunha and coworkers investigated femtosecond laser surface texturing as a method to reduce *Staphylococcus aureus* colonization and biofilm formation of Grade 2 titanium alloy surfaces. [26]

They found that laser microtexturing could confer antibacterial properties to the titanium surfaces when compared to the control, polished surfaces, and that the treatment caused a reduction in bacteria agglomeration, decreasing the tendency to form biofilms. While the latter finding can be confirmed by CLSM observation of the present *in vitro* study, the type of surface micro-topography obtained by Cunha and coworkers is markedly different from that of the present study, as well as the titanium alloy and the control surface that was employed.

In 2016, Di Giulio and coworkers compared 48 h *Porphyromonas gingivalis* biofilm formation on laser-treated surface with biofilm formation on grit-blasted and machined surfaces. [23] The laser-treated surface of titanium disks was the same as the one tested in our *in vitro* experiment. In agreement with our data, the authors concluded that the laser-treated surface was significantly less colonized than grit-blasted surfaces. Drago and coworkers, in the same year, studied the *in vitro* biofilm formation by *Staphylococcus aureus*, *Pseudomonas aeruginosa* and *Porphyromonas gingivalis* on disks of the same laser-treated surface and a grit-blasted surface. [24] Biofilm formation was significantly lower on the laser-treated surface than on the grit-blasted one for all the tested microorganisms.

It must be pointed out that these three studies were performed under static conditions of incubation and using monospecific biofilms. In our study, the use of a bioreactor allowed biofilm growth in a defined environment much closer to clinical conditions. The static conditions cannot reproduce the shear stresses that deeply affect biofilm development and structure when it grows exposed to a medium or saliva flow. This experimental setting could explain the differences between our data and those of Di Giulio and coworkers on machined surfaces. [23] Our data show that surface roughness of machined surfaces is significantly lower than that of laser-treated and grit-blasted surfaces. The influence of this parameter on biofilm formation could be enhanced by the presence of shear forces related to the medium flow.

A critical issue is the use of a preconditioning phase allowing the formation of a salivary pellicle on the specimens’ surface. The early colonization of titanium implant surfaces is not completely characterized, but the steps of microbial adherence and biofilm formation are probably similar to those occurring on the surfaces of natural hard tissues. [6,7,46] All oral surfaces, including titanium surfaces exposed to the oral environment, are covered by a thin film of adsorbed proteins originating from saliva. These provide a broad spectrum of potential receptors for the adherence of pioneer microorganisms. The absence of saliva preconditioning in the experiments of Di Giulio and coworkers [23] and Drago and coworkers [24] can probably explain the difference with our results.

Another crucial issue in employing a reductionist research approach to the study of oral biofilms is the use of a monospecific biofilm versus a complex oral microcosm model. The use of a single microorganism does not allow the *in vitro* reproduction of the complex interactions taking place in natural oral ecosystems, hence it is not a representative model of the microbial species colonizing the tested materials’ surface *in vivo*. Fresh whole saliva was used as an inoculum in the *in vitro* study to reproduce the complex supragingival flora, thus obtaining data about colonization of titanium surfaces when exposed to the oral environment as a consequence of peri-implantitis.

The results of our bioreactor study allowed the design of an *in situ* study to confirm the *in vitro* data. The study results showed that both laser-treated and machined surfaces exhibited significantly lower biofilm formation than grit-blasted surfaces. Zaugg and coworkers, evaluating the *in situ* brushing efficacy of a powered toothbrush on titanium disks, compared biofilm formation on laser-treated surfaces, machined surfaces and grit-blasted, acid etched, and chemically modified surfaces. [25] They demonstrated that laser-treated and grit-blasted surfaces showed significantly higher biofilm formation than machined surfaces. The difference between these results and those of the present study can be explained by some relevant differences in the setup as well as in the surface design. Zaugg and coworkers [25] used titanium disks with a flat surface instead of a threaded implant surface design. Additionally, the laser-treated surface showed parallel 7-micron deep grooves instead of regularly distributed 20-micron deep pits. Furthermore, in their tray design, the disks were placed with the treated surface against the palate, 2 or 3 mm from the mucosa surface, [25] whereas the specimens were directly exposed to salivary flow and to the cleaning action of buccal mucosa in the present study. Also, in our study, volunteers wore the tray for 48 h rather than 24 h, which probably allowed a more structured biofilm to be obtained.

Within the limitations of this study, related to the artificial environment produced *in vitro* to simulate oral conditions, and the lack of peri-implant soft tissues in the *in situ* experiment, the results of the present investigations can be promising from a translational point of view, allowing to better understand how to reduce biofilm formation on implant surfaces in the event of their exposure at the transmucosal level.

Nonetheless, further studies are needed to investigate biofilm formation on laser-treated surfaces by subgingival flora colonizing the peri-implant sulcus, providing useful information about the biological behavior of these surfaces at a subgingival level.

In conclusion, this study used an *in vitro* oral microcosm model grown using a bioreactor, and an *in situ* model based on intraoral trays, to evaluate biofilm formation by supragingival microbial species on a newly designed, laser microtextured titanium surface. The *in situ* results demonstrated that laser-created micro-topography can reduce biofilm formation, with a maximal effect when the surface is blasted orthogonally by the laser beam. However, when the beam blasts the titanium surface at a different angle, as on the inclined portion of the threads, this effect is lost. An accurate optimization of surface micro-topography is therefore paramount for the most effective prevention of biofilm formation. The *in vitro* bioreactor-based model provided useful viable biomass data which were highly comparable with those obtained in the oral environment. The *in vitro* set-up, however, did not show comparable biofilm morphology over orthogonally blasted surfaces when confronting CLSM data. Reasons for this behavior might be the differences in materials’ macro-morphology (disks vs. threads), the fact that subjects removed the trays and brushed their teeth with a fluoride-containing toothpaste, and a direct contact of *in situ* specimens with oral mucosa, which may all have contributed to the influence that the tested surfaces have had on biofilm formation.

## Supporting information

S1 Table Dataset is provided for the *in vitro* and *in situ* results of the MTT assay (OD values) as well as for surface roughness analysis (Ra values). (XLSX)

S2 Table Dataset is provided for the mean (left and right average) contact angles of the tested surfacesm from where surface free energy was calculated. Dataset is also provided for total surface coverage, and percentage of viable and dead bacteria in the biomass, resulting from CLSM MIP images that were processed using COMSTAT software.
(XLSX)

## References

[1] Zhao G, Schwartz Z, Wieland M, Rupp F, Geis‐Gerstorfer J, Cochran DL, et al High surface energy enhances cell response to titanium substrate microstructure. J Biomed Mater Res A. 2005;74(1): 49–58. 10.1002/jbm.a.30320 15924300

[2] Van Noort R. Titanium: the implant material of today. J Mater Sci. 1987;22(11): 3801–3811.

[3] Albrektsson T, Donos N. Working Group 1. Implant survival and complications. The Third EAO consensus conference. Clin Oral Implants Res. 2012;23: 63–5. 10.1111/j.1600-0501.2012.02557.x 23062128

[4] Lossdörfer S, Schwartz Z, Wang L, Lohmann CH, Turner JD, Wieland M, et al Microrough implant surface topographies increase osteogenesis by reducing osteoclast formation and activity. J Biomed Mater Res A. 2004;70(3): 361–369. 10.1002/jbm.a.30025 15293309

[5] Jimbo R, Sawase T, Baba K, Kurogi T, Shibata Y, Atsuta M. Enhanced initial cell responses to chemically modified anodized titanium. Clin Implant Dent Res. 2008;10(1): 55–61. 10.1111/j.1708-8208.2007.00061.x 18254741

[6] Norowski PA, Bumgardner JD. Biomaterial and antibiotic strategies for peri‐implantitis: A review. J Biomed Mater Res B Appl Biomater. 2009;88(2): 530–543. 10.1002/jbm.b.31152 18698626

[7] Gosau M, Hahnel S, Schwarz F, Gerlach T, Reichert TE, Bürgers R. Effect of six different peri-implantitis disinfection methods on *in vivo* human oral biofilm. Clin Oral Impl Res. 2010;21: 866–872. 10.1111/j.1600-0501.2009.01908.x 20666798

[8] Lang NP, Berglundh T. Periimplant diseases: where are we now? Consensus of the Seventh European Workshop on Periodontology. J Clin Periodontol. 2011;38(s11): 178–181. 21323713 10.1111/j.1600-051X.2010.01674.x

[9] Mombelli A, Müller N, Cionca N. The epidemiology of peri‐implantitis. Clin Oral Impl Res. 2012;23(s6): 67–76. 10.1111/j.1600-0501.2012.02541.x 23062130

[10] Khammissa RA, Feller L, Meyerov R, Lemmer J. Peri-implant mucositis and peri-implantitis: bacterial infection. South African Dental Journal. 2012;67: 70–74. 23189895

[11] Bürgers R, Gerlach T, Hahnel S, Schwarz F, Handel G, Gosau M. *In vivo* and *in vitro* biofilm formation on two different titanium implant surfaces. Clin Oral Implants Res. 2010;21(2): 156–164. 10.1111/j.1600-0501.2009.01815.x 19912269

[12] Berglundh T, Gotfredsen K, Zitzmann NU, Lang NP, Lindhe J. Spontaneous progression of ligature induced peri‐implantitis at implants with different surface roughness: an experimental study in dogs. Clin Oral Impl Res. 2007;18(5): 655–661. 10.1111/j.1600-0501.2007.01397.x 17608738

[13] Busscher HJ, Rinastiti M, Siswomihardjo W, van der Mei HC. Biofilm formation on dental restorative and implant materials. J Dent Res. 2010;89: 657–665. 10.1177/0022034510368644 20448246

[14] Holmberg KV, Abdolhosseini M, Li Y, Chen X, Gorr SU, Aparicio C. Bio-inspired stable antimicrobial peptide coatings for dental applications. Acta Biomater. 2013;9: 8224–8231. 10.1016/j.actbio.2013.06.017 23791670 PMC3758876

[15] He S, Zhou P, Wang L, Xiong X, Zhang Y, Deng Y, et al Antibiotic decorated titanium with enhanced antibacterial activity through adhesive polydopamine for dental/bone implant. J R Soc Interface. 2014;11: 20140169 10.1098/rsif.2014.0169 24647910 PMC4006258

[16] Allaker RP. The use of nanoparticles to control oral biofilm formation. J Dent Res. 2010;89(11): 1175–1186. 10.1177/0022034510377794 20739694

[17] van Hengel IA, Riool M, Fratila-Apachitei LE, Witte-Bouma J, Farrell E, Zadpoor AA, et al Selective laser melting porous metallic implants with immobilized silver nanoparticles kill and prevent biofilm formation by methicillin-resistant *Staphylococcus aureus*. Biomaterials. 2017;140: 1–5. 10.1016/j.biomaterials.2017.02.030 28622569

[18] Teughels W, Van Assche N, Sliepen I, Quirynen M. Effect of material characteristics and/or surface topography on biofilm development. Clin Oral Implants Res. 2006;17(S2): 68–81. 16968383 10.1111/j.1600-0501.2006.01353.x

[19] Renner LD, Weibel DB. Physicochemical regulation of biofilm formation. MRS bulletin. 2011;36(5): 347–355. 10.1557/mrs.2011.65 22125358 PMC3224470

[20] Lorenzetti M, Dogša I, Stosšicki T, Stopar D, Kalin M, Kobe S, et al The influence of surface modification on bacterial adhesion to titanium-based substrates. ACS Appl Mater Interfac. 2015;7(3): 1644–1651. 10.1021/am507148n 25543452

[21] Lepore S, Milillo L, Trotta T, Castellani S, Porro C, Panaro MA, et al Adhesion and growth of osteoblast-like cells on laser-engineered porous titanium surface: expression and localization of N-cadherin and beta-catenin. J Biol Regul Homeost Agents. 2013;27: 531–541. 23830402

[22] Cardelli P, Cecchetti F, Montani M, Bramanti E, Arcuri C. Clinical assessment of submerged vs non-submerged implants placed in pristine bone. Oral Implantol. 2014;6: 89–93. PMC4051271 24971162

[23] Di Giulio M, Traini T, Sinjari B, Nostro A, Caputi S, Cellini L. Porphyromonas gingivalis biofilm formation in different titanium surfaces, an in vitro study. Clin Oral Impl Res. 2016;27: 918–925. 10.1111/clr.12659 26249670

[24] Drago L, Bortolin M, De Vecchi E, Agrappi S, Weinstein RL, Mattina R, et al Antibiofilm activity of sandblasted and laser-modified titanium against microorganisms isolated from peri-implantitis lesions. J Chemother. 2016;28(5): 383–389. 10.1080/1120009X.2016.1158489 27240314

[25] Zaugg LK, Astasov-Frauenhoffer M, Braissant O, Hauser-Gerspach I, Waltimo T, Zitzmann N. Determinants of biofilm formation and cleanability of titanium surfaces. Clin Oral Impl Res. 2017;28: 469–475. 10.1111/clr.12821 26992098

[26] Cunha A, Elie AM, Plawinski L, Serro AP, do Rego AM, Almeida A, et al Femtosecond laser surface texturing of titanium as a method to reduce the adhesion of *Staphylococcus aureus* and biofilm formation. Appl Surf Sci. 2016;360: 485–493.

[27] Fadeeva E, Truong VK, Stiesch M, Chichkov BN, Crawford RJ, Wang J, et al Bacterial retention on superhydrophobic titanium surfaces fabricated by femtosecond laser ablation. Langmuir. 2011;27(6): 3012–3019. 10.1021/la104607g 21288031

[28] Patil D, Aravindan S, Wasson MK, Vivekanandan P, Rao PV. Fast Fabrication of Superhydrophobic Titanium Alloy as Antibacterial Surface Using Nanosecond Laser Texturing. J Micro Nano Manufactur. 2018;6(1): 011002.

[29] Berardi D, Colagiovanni M, Scoccia A, Raffaelli LU, Manicone PF, Perfetti G. Evaluation of a new laser surface implant: scanning electron microscopy/energy dispersive X-ray and X-ray photoelectron spectroscopy analyses. J Biol Regul Homeost Agents. 2008;22(3): 161–167. 18842169

[30] Ionescu AC, Hahnel S, Cazzaniga G, Ottobelli M, Braga RR, Rodrigues MC, et al *Streptococcus mutans* biofilm formation on experimental composites containing dicalcium phosphate dihydrate nanoparticles. J Mater Sci Mater Med. 2017;28(108): 1–11. 28540581 10.1007/s10856-017-5914-7

[31] Ionescu AC, Brambilla E, Wastl DS, Giessibl FJ, Cazzaniga G, Schneider-Feyrer S, et al Influence of matrix and filler fraction on biofilm formation on the surface of experimental resin-based composites. J Mater Sci Mater Med. 2015;26(1): 5372 10.1007/s10856-014-5372-4 25604698

[32] McBain AJ, Bartolo RG, Catrenich CE, Charbonneau D, Ledder RG, Gilbert P. Growth and molecular characterization of dental plaque microcosms. J Appl Microbiol. 2003;94(4): 655–664. 12631201 10.1046/j.1365-2672.2003.01876.x

[33] Hahnel S, Ionescu AC, Cazzaniga G, Ottobelli M, Brambilla E. Biofilm formation and release of fluoride from dental restorative materials in relation to their surface properties. J Dent. 2017;60: 14–24. 10.1016/j.jdent.2017.02.005 28212980

[34] World Medical Association. World Medical Association Declaration of Helsinki: ethical principles for medical research involving human subjects. J Am Med Assoc. 2013;310(20): 2191–2194. 10.1001/jama.2013.281053 24141714

[35] O’Leary TJ, Drake RB, Naylor JE. The plaque control record. J Periodontol. 1972; 43:38 10.1902/jop.1972.43.1.38 4500182

[36] Ainamo J, Bay I. Problems and proposals for recording gingivitis and plaque. Inter Dent J. 1975;25: 229–235. 1058834

[37] Warnakulasuriya S, Dietrich T, Bornstein MM, Peidró EC, Preshaw PM, Walter C, et al Oral health risks of tobacco use and effects of cessation. Inter Dent J. 2010;60(1): 7–30. 20361572

[38] Truong VK, Lapovok R, Estrin YS, Rundell S, Wang JY, Fluke CJ, et al The influence of nano-scale surface roughness on bacterial adhesion to ultrafine-grained titanium. Biomaterials. 2010;31(13): 3674–3683. 10.1016/j.biomaterials.2010.01.071 20163851

[39] Schwarz F, Sculean A, Wieland M, Horn N, Nuesry E, Bube C, et al Effects of hydrophilicity and microtopography of titanium implant surfaces on initial supragingival plaque biofilm formation. A pilot study. Mund-, Kiefer-und Gesichtschirurgie. 2007;11(6): 333–338. 10.1007/s10006-007-0079-z 17940813

[40] Cazzaniga G, Ottobelli M, Ionescu A, Garcia-Godoy F, Brambilla E. Surface properties of resin-based composite materials and biofilm formation: A review of the current literature. Am J Dent. 2015;28(6): 311–320. 26846036

[41] Bollen CM, Lambrechts P, Quirynen M. Comparison of surface roughness of oral hard materials to the threshold surface roughness for bacterial plaque retention: a review of the literature. Dent Mater. 1997;13(4): 258–269. 11696906 10.1016/s0109-5641(97)80038-3

[42] Cazzaniga G, Ottobelli M, Ionescu AC, Paolone G, Gherlone E, Ferracane JL, et al In vitro biofilm formation on resin-based composites after different finishing and polishing procedures. J Dent. 2017;67: 43–52. 10.1016/j.jdent.2017.07.012 28750776

[43] Machado MC, Cheng D, Tarquinio KM, Webster TJ. Nanotechnology: pediatric applications. Pediatr Res. 2010;67(5): 500 10.1203/PDR.0b013e3181d68e78 20139795

[44] Anselme K, Davidson P, Popa AM, Giazzon M, Liley M, Ploux L. The interaction of cells and bacteria with surfaces structured at the nanometre scale. Acta biomaterialia. 2010;6(10): 3824–3846. 10.1016/j.actbio.2010.04.001 20371386

[45] Vorobyev AY, Guo C. Direct femtosecond laser surface nano/microstructuring and its applications, Laser Photon Rev. 2013;7: 385–407.

[46] Leonhardt A, Renvert S, Dahlen G: Microbial findings at failing implants. Clin Oral Implants Res. 1999;10: 339–345. 10551058 10.1034/j.1600-0501.1999.100501.x

